# Identification of biomarkers of immune checkpoint blockade efficacy in recurrent or refractory solid tumor malignancies

**DOI:** 10.18632/oncotarget.27466

**Published:** 2020-02-11

**Authors:** Richard K. Yang, Yun Qing, Fatima Zahra Jelloul, Mark J. Routbort, Peng Wang, Kenna Shaw, Jiexin Zhang, Jack Lee, L. Jeffrey Medeiros, Scott Kopetz, Michael T. Tetzlaff, Russell R. Broaddus

**Affiliations:** ^1^ Department of Hematopathology, University of Texas MD Anderson Cancer Center, Houston, TX, USA; ^2^ Department of Biostatistics, University of Texas MD Anderson Cancer Center, Houston, TX, USA; ^3^ Institute for Personalized Cancer Treatment (IPCT), University of Texas MD Anderson Cancer Center, Houston, TX, USA; ^4^ Department of Bioinformatics and Computational Biology, University of Texas MD Anderson Cancer Center, Houston, TX, USA; ^5^ Department of Gastrointestinal Medical Oncology, University of Texas MD Anderson Cancer Center, Houston, TX, USA; ^6^ Departments of Anatomical Pathology and Translational Molecular Pathology, University of Texas MD Anderson Cancer Center, Houston, TX, USA; ^7^ Department of Pathology and Laboratory Medicine, University of North Carolina at Chapel Hill, Chapel Hill, NC, USA

**Keywords:** biomarkers, immunotherapy, mutations, TMB, histology

## Abstract

Patients with advanced solid malignancies recurrent or resistant to standard therapy have limited treatment options. The role of molecular biomarkers for predicting immune checkpoint blockade (ICB) efficacy are not well characterized in these patients. Tumor mutational profiles of 490 patients with a variety of advanced solid tumors enrolled in a prospective protocol were analyzed to identify prognostic and predictive biomarkers. ICB therapy was defined as treatment with any CTLA-4, PD-1, and/or PD-L1 monoclonal antibody. ICB treatment was associated with significantly improved overall survival compared to non-ICB therapy. Multivariate regression analysis *including the two variables* of tumor mutation burden (TMB) and ICB, and their interaction term, showed favorable survival associated with ICB, unfavorable survival associated with TMB without ICB treatment, and improved outcome with increasing TMB in ICB treated patients. Tumor *TP53* mutation was associated with worse survival, but these patients still benefitted from ICB. A more comprehensive multivariate analysis including cancer type, specific gene mutations, and TMB revealed that ICB treatment was an independent predictor of improved overall survival. Therefore, ICB-based therapeutic trials are beneficial in patients with advanced solid malignancies, but the most benefit may be restricted to patients with the right combination of TMB and specific tumor histology and genotype.

## INTRODUCTION

Patients with advanced cancers who are initially resistant to frontline therapy or who develop recurrent disease have a finite survival [1], and choosing the right therapy within this window is crucial. We previously demonstrated that matched targeted therapy in this patient population is associated with better survival, but this benefit was realized for a small subset of patients [2]. One alternative approach to consider for this patient population may be the use of immune checkpoint blockade (ICB), which has changed therapeutic strategies for the treatment of a number of solid tumors [3], especially non-small cell lung carcinoma, melanoma, and Merkel cell carcinoma [4–6]. When faced with patients with a variety of different advanced cancer types, it can be difficult to decide which specific patients should be treated with ICB.

In a prospective trial reported previously, a large next-generation sequencing (NGS) panel comprised of the entire coding regions of 409 cancer-related genes was employed to identify clinically actionable gene amplifications or mutations to help place patients onto matched targeted therapy trials [2]. Another potential use for such larger NGS panels is calculation of tumor mutation burden (TMB), a promising biomarker for prediction of response to immune checkpoint blockade [7]. TMB has been shown to predict response to ICB in patients with non-small cell lung carcinoma (NSCLC) [8, 9], melanoma [10]**,** gastrointestinal (GI) and endometrial adenocarcinomas [11]**,** as well as histology agnostic solid tumors [12]. It remains unclear what predictive and/or prognostic significance TMB has in patients with advanced stage, recurrent or treatment refractory solid malignancies.

Recently, patients with tumor mutations in certain DNA damage repair (DDR) genes such as POLE [13], base excision repair (BER) genes [14], and mismatch repair (MMR) genes [9] have been shown to respond to ICB. However, the potential ICB response in patients with tumor mutations in other DDR pathway genes is not yet well characterized. Recent literature proposes that certain non-small cell carcinomas with mutations in TP53 may have favorable response to ICB [15]. However, the effects of TP53 mutation as a modifier of ICB response is also not yet well described.

During the initial recruitment phase for this prospective protocol (2014–2015), the efficacy of ICB for melanoma and a few other cancer types was just beginning to be evaluated in clinical trials [16–19]. Therefore, ICB treatment of patients with higher TMB was not considered a matched targeted therapy for the trial [2]. During the course of retrospective review of the trial data, we noted that a number of patients were treated with ICB after enrollment onto the trial. We therefore wanted to determine if immunotherapy had utility in this difficult to treat patient population. We also sought to identify what clinical variables, if any, were associated with ICB treatment response. In this report, we describe a number of modifying factors that may be useful in the decision to treat with immune checkpoint blockade patients who have advanced solid malignancies that are recurrent or refractory to frontline therapies.

## RESULTS

**Figure 1:**
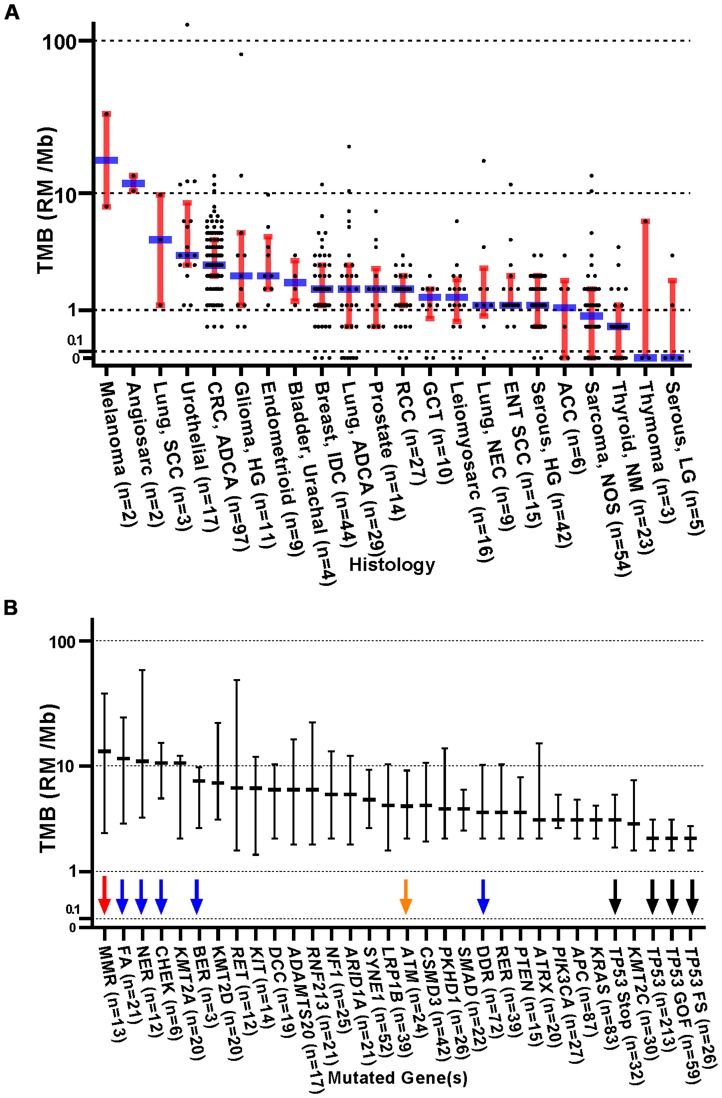
Distribution of TMB (RMs /Mb) stratified by original histologic diagnosis and by gene mutation profile. (**A**) Dot plots with medians (blue) and interquartile ranges (red). Abbreviations: ADCA: Adenocarcinoma; ACC: Adrenal Cortical Carcinoma; ENT: Ear Nose Throat; GCT: Germ cell Tumor; LG: Low Grade; HG: High grade; IDC: Invasive Ductal Carcinoma; NEC: Neuroendocrine Carcinoma; NM: Non-Medullary Thyroid Carcinoma; NOS: Not Otherwise Specified; RCC: Renal Cell Carcinoma; SCC: Squamous Cell Carcinoma. Since response to ICB was not interrogated in these relationships, all 554 patients were included in this analysis, regardless of treatment status. (**B**) Distribution of TMB (RMs /Mb) stratified by individual somatic gene mutation. Tumors with somatic mutations within these genes or gene panels were plotted against a standardized TMB logarithmic scale with medians and interquartile ranges shown. The red arrow highlights the relatively high TMB seen in mismatch repair (MMR) gene mutated tumors. The blue arrows indicate tumors with mutations in other DNA repair pathways. The orange arrow indicates ATM mutated tumors. TP53 mutated tumors demonstrated relatively lower TMB (black arrows). Both stratifications yield a *p* < 0.0001 in one-way ANOVA analysis. TMB - Tumor Mutation Burden; RM - Reported Mutations; Mb – Megabase; DDR (DNA Damage Repair) = BER, CHEK, FA, MMR, NER, RER. MMR (Mismatch Repair) = *MLH1*, *MSH2*, *MSH6*, *PMS1*, *PMS2*; RER (Recombination Repair) = *ATM*, *ATR*, *XRCC2*, *RAD50*, *WRN*, *PARP1*, *NBN*, *MRE11A*; FA (Fanconi anemia pathway) = *FANCA*, *FANCC*, *FANCD2*, *FANCF*, *FANCG*, *PALB2*, *BRIP1*; NER (Nucleotide Excision Repair) = *ERCC2*, *4*, *5*, *XPC*, *XPA*; *BER* (Base Excision Repair) = *SMUG1*, *MUTYH*; *CHEK* (Checkpoint Kinases) = *CHEK1*, *CHEK2*. Since response to ICB was not interrogated in these relationships, all 554 patients were included in this analysis, regardless of treatment status.

Of the 554 patients consented for study, 32 did not receive any treatment on study, and 32 patients were lost to follow up following enrollment. Therefore, all analyses are focused on 490 patients who received some type of treatment while on study, all of whom had AJCC 8^th^ edition stage III or IV disease (or neurologic tumors of WHO grade III or IV). A wide variety of cancer types were represented in this study (Table 1 and Supplementary Tables 1 and 2). Notably, only two melanoma patients were enrolled on this protocol. The mean number of somatic mutations per tumor was 5.4 (median of 3, range 0–221). Table 1 summarizes the distribution of patient tumors by cancer type, number of reported mutations, and whether ICB was employed as a treatment while on study. Low TMB was relatively common in patients with adenoid cystic carcinoma of the salivary glands and non-medullary thyroid carcinoma. In contrast, cancers such as colorectal carcinoma, urothelial carcinoma, head and neck squamous cell carcinoma, endometrial carcinoma, and germ cell tumors (GCTs) rarely had low TMB, showing fewer than 10% of total cases having 0–1 mutations (Table 1). The relationship between histology and TMB was comparable to previous reports (Figure 1A), demonstrating high TMB in melanoma, squamous cell carcinoma of the lung, and urothelial carcinoma as well as low TMB in non-medullary thyroid carcinoma, thymoma and low grade serous gynecologic carcinoma. In comparison, when stratifying TMB by mutated gene or pathway, tumors with mutated DNA repair genes demonstrated relatively increased TMB (red, blue, and orange arrows, Figure 1B), whereas TP53 mutated tumors tended to demonstrate relatively lower TMB (black arrows).

**Table 1:** Distribution of tumor mutational burden and ICB treatment by cancer type Since response to ICB was interrogated in these relationships, only the 490 treated patients were included in this analysis

			Reported Mutations (*n*; %)						Treatment (*n*; %)			
	Patients (*n*, %)		0–1		2–12		>12		ICB		Non-ICB	
**Cancer Type**												
Colorectal Adenocarcinoma	87	17.8	2	2.3	80	92.0	5	5.7	5	5.7	82	94.3
Sarcoma, High Grade	64	13.1	26	40.6	36	56.3	2	3.1	13	20.3	51	79.7
Breast Carcinoma	38	7.8	7	18.4	30	78.9	1	2.6	2	5.3	36	94.7
Serous Carcinoma, High Grade	37	7.6	10	27.0	27	73.0	0	0.0	0	0.0	37	100.0
Non-Colorectal GI Carcinoma	33	6.7	10	30.3	21	63.6	2	6.1	6	18.2	27	81.8
Non-Small Cell Lung Carcinoma	29	5.9	8	27.6	18	62.1	3	10.3	15	51.7	14	48.3
Renal Cell Carcinoma	28	5.7	8	28.6	20	71.4	0	0.0	16	57.1	12	42.9
Thyroid carcinoma	20	4.1	14	70.0	6	30.0	0	0.0	4	20.0	16	80.0
Adenoid Cystic Carcinoma	17	3.5	12	70.6	5	29.4	0	0.0	9	52.9	8	47.1
Urothelial Carcinoma	15	3.1	0	0.0	11	73.3	4	26.7	5	33.3	10	66.7
Head & Neck Squamous	12	2.4	1	8.3	10	83.3	1	8.3	4	33.3	8	66.7
Prostate Adenocarcinoma	12	2.4	4	33.3	7	58.3	1	8.3	3	25.0	9	75.0
Glioma, High Grade	9	1.8	2	22.2	5	55.6	2	22.2	0	0.0	9	100.0
Endometrioid Carcinoma	7	1.4	0	0.0	6	85.7	1	14.3	0	0.0	7	100.0
Germ Cell Tumor	5	1.0	0	0.0	5	100.0	0	0.0	0	0.0	5	100.0
Other	77	15.7	30	39.0	39	50.6	8	10.4	21	27.3	56	72.7
**Overall**	490	100	134	27.3	326	66.5	30	6.1	103	21.0	387	79.0

In 31 (6.3%) patients, PD-L1 expression was assessed by immunohistochemistry on a wide variety of tumors; 5 (16.1%) tumors were positive, defined as expression in >1% of tumor cells. Mismatch repair (MMR) was assessed by immunohistochemistry in 21 (4.3%) patients, 2 (9.5%) of whom showed MMR deficiency. PCR-based microsatellite instability (MSI) was performed on 20 (4.1%) patient tumors 2 of which were MSI-High. Most of the MMR and MSI assessments were performed for patients with colorectal adenocarcinoma. ICB treatment was not significantly associated with tumor PD-L1 positivity (*p* > 0.99), presence of tumor MSI-high (*p* = 0.10), or presence of MMR deficiency (*p* = 0.43) (data not shown).

**Figure 2:**
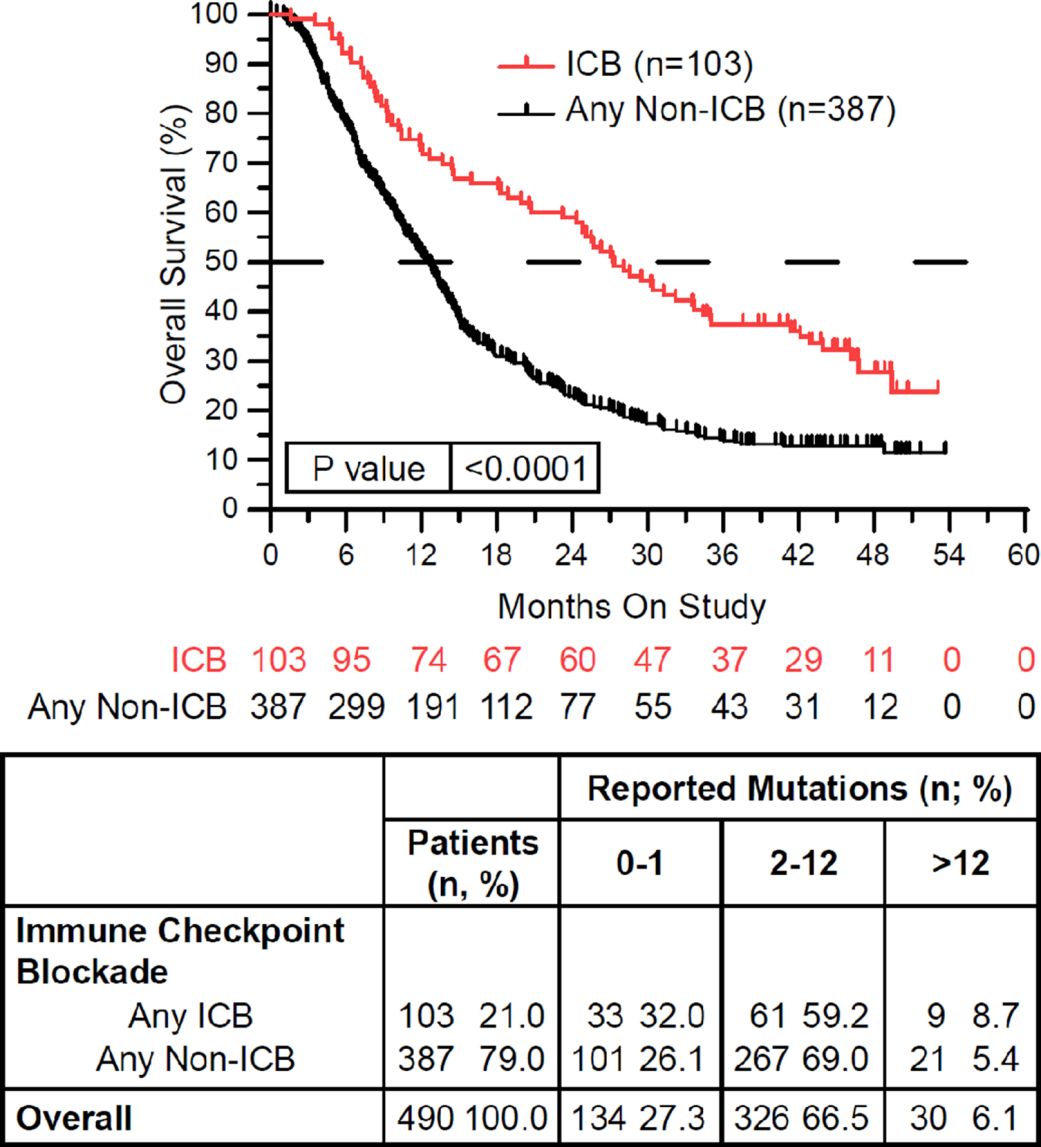
Immune checkpoint blockade (ICB) therapy is associated with improved overall survival. Patients treated with ICB while on study (red) demonstrated improved overall survival compared to patients treated with any other non-ICB therapy while on study (black) (HR = 0.542, 95%CI [0.436 to 0.675], *p* < 0.0001). In the above tables, 0–1 RM (reported mutations) is equivalent to 0–0.573/Mb tumor mutation burden (TMB), 2–12 RM (reported mutations) is equivalent to 1.146–6.877/Mb TMB, and >12 RM (reported mutations) is equivalent to >6.88/Mb TMB. Since response to ICB was interrogated in these relationships, only the 490 treated patients were included in this analysis.

One hundred and three of 490 (21%) treated patients received ICB while on study (Table 1). ICB treatment was associated with significantly longer overall survival (*p* < 0.0001) (Figure 2). ICB was most commonly attempted in patients with non-small cell lung carcinoma and renal cell carcinoma, and adenoid cystic carcinoma. In contrast, ICB was given to fewer than 10% of patients with colorectal cancer, breast carcinoma, high grade serous carcinoma, glioma, endometrial carcinoma, and germ cell tumors. None of these ICB treated patients had melanoma. Of the 103 ICB treated patients, 18 (17.4%) were tested for PD-L1 IHC expression, MMR deficiency, and/or MSI instability. Four (3.9%) patients were found to be PD-L1 IHC positive (>1%) or had MMR deficient or MSI-High tumors (data not shown). The remaining patients were treated with ICB empirically.

**Figure 3:**
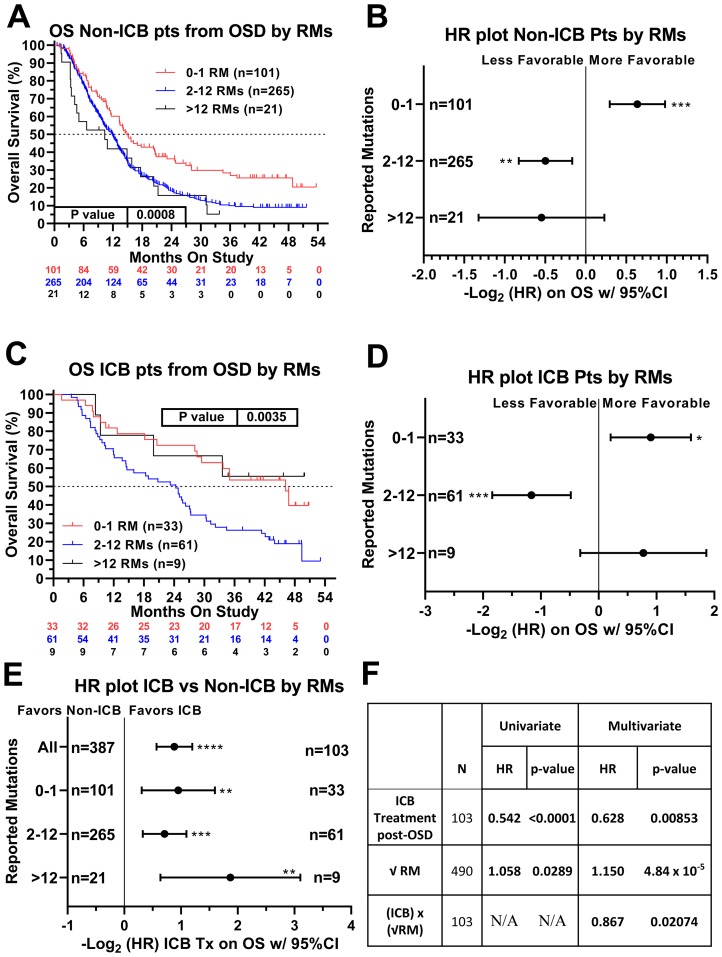
Relationship of ICB treatment and tumor mutation burden on overall survival. When examining patients who never received ICB, higher TMB was associated with worse overall survival (**A** and **B**). In contrast, in patients who received ICB therapy, the lower and upper extremes of TMB were associated with better overall survival (**C** and **D**). Patients with TMB>12 demonstrated the most benefit when treated with an ICB regimen (**E**). Panel (**F**) summarizes the univariate and multivariable regression of the effects of ICB therapy, the square root transformation of reported mutations (RM), and the interaction between these two variables. In both the univariate and multivariate analyses, ICB therapy was associated with favorable survival and the square root of RM was associated with unfavorable survival. The interaction between these two variables (that is, increasing RM in the setting of ICB treatment) was associated with favorable survival. Hazard ratios of the three TMB groups were compared internally to all other patients within this patient cohort. HRs are –Log2 transformed so that a HR of 2 equals 1.0 and a HR of 0.5 equals -1.0. ^****^ *p*-value < 0.0001; ^***^ *p*-value < 0.001; ^**^ *p*-value < 0.01, ^*^ *p*-value < 0.05. In the above charts and tables, 0–1 RM (reported mutations) is equivalent to 0–0.573/Mb tumor mutation burden (TMB). 2–12 RM (reported mutations) is equivalents to 1.146–6.877/Mb TMB and >12 RM (reported mutations) is equivalent to >6.88/Mb TMB. Since response to ICB was interrogated in these relationships, only the 490 treated patients were included in this analysis.

We next sought to identify factors that were associated with ICB treatment response and/or resistance. Recursive partitioning for classification and tree methods (described in the methods section) were used to determine the optimal cut-points for TMB, which were the following three groups: 0–1 mutation, *n* = 134; 2–12 mutations, *n* = 326; and 13–221 mutations, *n* = 30. In patients not receiving ICB, there was a continuous decrement of survival with increasing TMB (*p* = 0.0008 Log-rank (Mantel-Cox) test, *p* = 0.0002 Log rank test for trend) (Figure 3A). Patients not receiving ICB with low TMB (0–1 RMs) had significantly better survival compared with the remainder of the non-ICB treated patients (HR = 0.643, 95% CI [0.508–0.815]) (Figure 3A, 3B). Among ICB treated patients, however, a different pattern emerged with patients at the extreme ends of the TMB spectrum having relatively favorable outcomes. Again, patients with low TMB (0–1 reported mutations) demonstrated better relative survival (HR = 0.535, 95% CI [0.331–0.866]) (Figure 3C and 3D). Patients with high TMB (>12 reported mutations), however, showed a trend towards improved survival when treated with ICB (HR = 0.586, 95% CI [0.275–1.246]) (Figure 3C and 3D) (*p* = 0.0035 Log-rank (Mantel-Cox) test, *p* = 0.211 Log rank test for trend). Patients with high TMB tumors exhibited the greatest benefit when treated with ICB (HR = 0.273, 95% CI [0.116–0.643]) (Figure 3E and Supplementary Figure 1). Univariate analysis using Cox proportional hazards model demonstrated that ICB therapy was associated with significantly improved survival (HR = 0.542, 95% CI [0.436–0.675], *p* < 0.0001). Increasing TMB (expressed as the square root of reported mutations) was associated with poorer survival (HR = 1.058, *p* = 0.0289, Figure 3F). An interaction term between ICB therapy and the square root of reported tumor mutations in the multivariate analysis revealed that these two variables were not independent (*p* = 0.0207). Rather, increasing number of mutations appeared to have a favorable association with survival when patients were treated with ICB (Figure 3F). For non-ICB treated patients, the HR associated with 1 unit increase in the square root of TMB was 1.150 (95% CI: 1.075–1.230, *p* < .0001), while for ICB treated patients, the HR associated with 1 unit increase in the square root of TMB was not statistically significant (0.997 [95% CI: 0.901–1.102, *p* = 0.946]). In the multivariate model, the estimates for the coefficients of ICB treatment, the square root of reported tumor mutations, and an interaction term between ICB therapy and the square root of reported tumor mutations were -0.465 (*p* = 0.0085), 0.140 (*p* < 0.0001), and −0.143 (*p* = 0.0207), respectively. To summarize, initial multivariate analysis demonstrated that both ICB treatment and TMB were both correlated with overall survival, and that there appeared to be an interaction between these two variables.

We next determined if any specific cancer types were over-represented in the highest TMB group. Of the 490 patients who received treatment while on study, 30 (6.1%) had tumors in the group with the highest TMB (>12 mutations). These 30 patients had a wide variety of different cancer types (Table 2), suggesting that cancer type was not necessarily an accurate predictor of high TMB and response to ICB. Interestingly, five of nine patients (55.5%) who received ICB are still alive as of January 2019, whereas 19 of 21 (90.5%) patients who did not receive ICB have died of disease.

**Table 2:** Summary of patients with highest TMB in study cohort

Primary site and diagnosis	Reported mutations	Treatment with immune checkpoint blockade	Death from disease
Glioma, High Grade, Frontal Lobe, Brain	143	No	Yes
Melanoma, Cutaneous	59	No	Yes
Endometrial Endometrioid Adenocarcinoma	46	No	Yes
Endometrial Carcinoma, Undifferentiated	43	No	Yes
Lung Small Cell Carcinoma	29	No	Yes
Optic Nerve Pilocytic Astrocytoma	23	No	Yes
Colorectal Adenocarcinoma	23	No	Yes
Bladder Urothelial Carcinoma Cell Bladder	21	No	Yes
Breast Adenocarcinoma	20	No	No
Bladder Urothelial Carcinoma	20	No	Yes
Nasal Squamous Cell Carcinoma	20	No	Yes
Angiosarcoma, Scalp	18	No	Yes
Colorectal Adenocarcinoma	18	No	Yes
Endometrial Adenocarcinoma, Endometrioid	17	No	Yes
Colorectal Adenocarcinoma	14	No	Yes
Melanoma of Unknown Primary	14	No	Yes
Small Intestinal Adenocarcinoma	14	No	Yes
Colorectal Adenocarcinoma	13	No	No
Prostatic Adenocarcinoma	13	No	Yes
Small Intestinal Adenocarcinoma	13	No	Yes
Lung Adenocarcinoma with Small Cell Features	13	No	Yes
Urothelial Carcinoma of Kidney, Micropapillary	221	Tremelimumab, Durvalumab	Yes
Squamous Carcinoma, Cutaneous	114	Nivolumab	No
Pituitary Gland Adenocarcinoma	76	Pembrolizumab	No
Lung Adenocarcinoma	36	Nivolumab, Durvalumab	Yes
Angiosarcoma, Scalp	23	Pembrolizumab	No
Bladder Urothelial Carcinoma	21	Nivolumab	No
Colorectal Adenocarcinoma	20	Nivolumab	Yes
Lung Squamous Cell Carcinoma	17	Ipilimumab	Yes
Parathyroid Adenocarcinoma	13	Pembrolizumab	No

**Figure 4:**
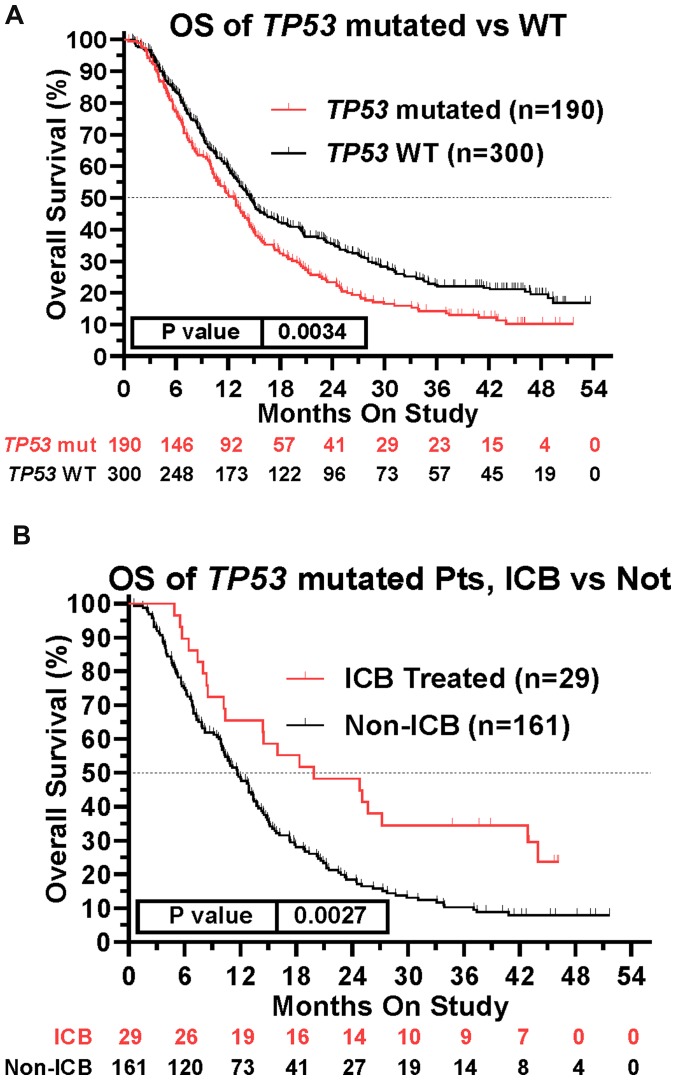
Impact of TP53 tumor mutation on survival and ICB treatment. (**A**) TP53 mutation was associated with worse survival across the patient cohort (HR = 1.367, 95%CI [1.109 to 1.686], *n* = 490, *p* = 0.0034, Figure 4A). (**B**) Retained efficacy of ICB in TP53 mutated tumors. The HR of ICB treatment in TP53 mutated patients (HR = 0.565, 95%CI [0.389 to 0.821], *n* = 190, *p* = 0.0027, Figure 4B) was comparable to the HR in patients with TP53 wildtype tumors (HR = 0.543, 95%CI [0.414 to 0.718], *n* = 300, *p* < 0.0001, Figure 5). Since response to ICB was interrogated in these relationships, only the 490 treated patients were included in this analysis.

**Figure 5:**
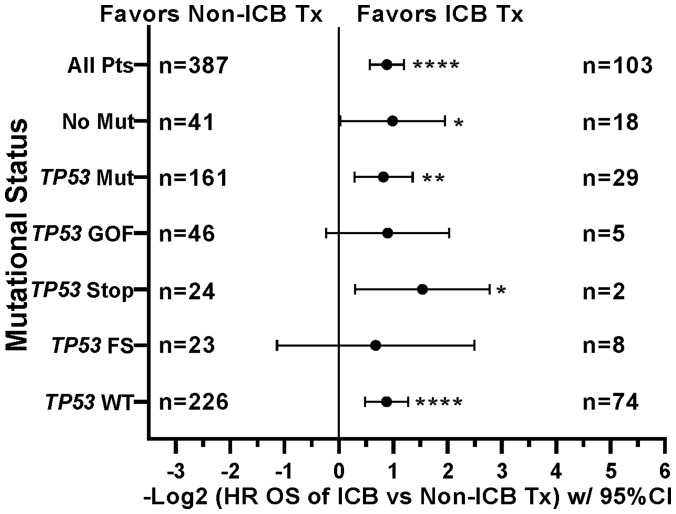
Tumors with mutations in TP53 demonstrate improved overall survival when treated with icb, similar to tp53 wildtype tumors. Tree plot of hazard ratios demonstrates retained efficacy of ICB in TP53 mutated tumors. The HR of ICB treatment in TP53 mutated patients (HR = 0.565, 95%CI [0.389 to 0.821], *n* = 190, *p* = 0.0027) was comparable to the HR in patients with TP53 wildtype tumors (HR = 0.543, 95%CI [0.414 to 0.718], *n* = 300, *p* < 0.0001). This suggested that presence of TP53 Mutation is not a contra-indication to ICB Treatment. HRs are –Log2 transformed so that a HR of 2 equals 1.0 and a HR of 0.5 equals -1.0. ^****^ *p*-value < 0.0001; ^***^ *p*-value < 0.001; ^**^ *p*-value < 0.01, ^*^ *p*-value < 0.05, ^#^ *p*-value < 0.10. Since response to ICB was interrogated in these relationships, only the 490 treated patients were included in this analysis. HRs for groups with small numbers of patients are provided for completeness only.

**Figure 6:**
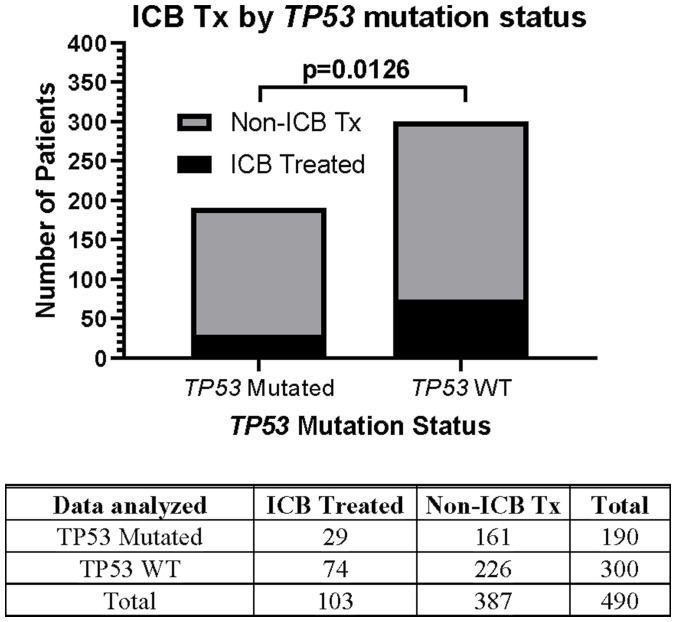
Relationship between *TP53* mutation status and likelihood of ICB therapy. Only 29/190 (15.3%) *TP53*-mutated patients were treated with ICB, a significantly lower proportion compared to that of *TP53* wild type patients 74/300 (24.7%). The Fisher’s exact test statistic yields a *p*-value of 0.0126. Since response to ICB was interrogated in these relationships, only the 490 treated patients were included in this analysis.

This prospective protocol specifically enrolled cancer patients with recurrent or advanced disease in which front-line therapy had failed. *TP53* mutations were the most common somatic mutation in this patient cohort (190/490 [38.8%] patients; Table 3), so we investigated whether *TP53* mutations were prognostic. Across all treated patients, *TP53* mutation was associated with a statistically significant decrease in overall survival (Figure 4A). Patients with *TP53* mutated tumors, however, still realized the survival benefit of ICB treatment (Figure 4B). In fact, the HR of ICB treatment in *TP53* mutated patients (HR = 0.565, 95%CI [0.389 to 0.821], *n =* 190, *p =* 0.0027, Figure 4B) versus that in *TP53* wild type patients (HR = 0.543, 95%CI [0.414 to 0.718], *n* = 300, *p* < 0.0001) was comparable (Figure 5). Therefore, the presence of a *TP53* mutation should not, by itself, discourage ICB treatment. However, only 29/190 (15.3%) TP53-mutated patients were treated with ICB, a significantly lower proportion compared to that of TP53 wild type patients (74/300 [24.7%]; Fisher Exact test *p* = 0.0126, Figure 6).

**Table 3:** Multivariate analysis for overall survival in study cohort of 490 treated patients Since response to ICB was interrogated in these relationships, only the 490 treated patients were included in this analysis

	N	Univariate		Multivariate	
		HR	*p*-value	HR	*p*-value
**Female**	256	0.868	0.1644	N/A	N/A
**ICB Treatment**	103	**0**.**542**	**<0**.**0001**	**0**.**621**	**0**.**01520**
**Cancer Type**					
Colorectal Carcinoma (CRC)	87	**1**.**858**	**<0**.**0001**	1.375	0.12947
Sarcoma, High Grade	64	0.776	0.0789	**0**.**663**	**0**.**02481**
Breast Carcinoma	38	1.145	0.5014	N/A	N/A
Serous Carcinoma, High Grade	37	**1**.**602**	**0**.**0249**	1.073	0.74109
Non-CRC Gastrointestinal	33	**1**.**615**	**0**.**0444**	1.358	0.16198
Non-Small Cell Lung	29	1.144	0.5319	N/A	N/A
Renal Cell Carcinoma	28	1.186	0.4428	N/A	N/A
Non- Medullary Thyroid	20	**0**.**466**	**0**.**0003**	**0**.**265**	**0**.**00087**
Adenoid Cystic Carcinoma	17	**0**.**500**	**0**.**0009**	**0**.**368**	**0**.**00513**
Urothelial Cell Carcinoma	15	0.983	0.9517	N/A	N/A
Head & Neck Squamous	12	1.644	0.1809	1.222	0.54366
Prostate Carcinoma	12	1.193	0.6112	N/A	N/A
Glioma	9	1.424	0.3932	N/A	N/A
Endometrial Carcinoma	7	1.086	0.8352	N/A	N/A
Germ Cell Tumors	5	3.353	0.1044	1.572	0.38098
Other	77	**0**.**702**	**0**.**0057**	**0**.**619**	**0**.**00504**
**Mutated Gene**					
*TP53*	190	**1**.**367**	**0**.**0034**	0.838	0.20043
*TP53* Gain of Function	51	**1**.**837**	**0**.**0013**	**1**.**372**	0.08231
*TP53* Frame Shift	23	**1**.**808**	**0**.**0298**	1.283	0.30556
*TP53* Stop Codon	32	0.809	0.2749	N/A	N/A
*APC*	80	**1**.**515**	**0**.**0048**	0.861	0.51652
*KRAS*	75	1.275	0.0962	0.726	0.06485
*LRP1B*	35	**1**.**768**	**0**.**0144**	**1**.**709**	**0**.**01308**
*PIK3CA*	25	**2**.**371**	**0**.**0022**	**1**.**531**	0.05730
*NF1*	22	1.217	0.4431	N/A	N/A
*ATM*	21	0.850	0.4893	N/A	N/A
*SMAD4*	21	1.648	0.0789	1.220	0.44884
*ARID1A*	19	1.061	0.8169	N/A	N/A
**Reported Mutations**					
-0–1 RM	134	**0**.**613**	**<0**.**0001**	1.305	0.50289
-2–12 RM	326	**1**.**593**	**<0**.**0001**	1.487	0.21770
>12 RM	30	0.961	0.8501	N/A	N/A
Sqrt RMs	***490***	**1**.**058**	**0**.**0289**	1.083	0.28354
**(ICB) x (√RM)**	103	***N/A***	***N/A***	0.931	0.33880

**Figure 7:**
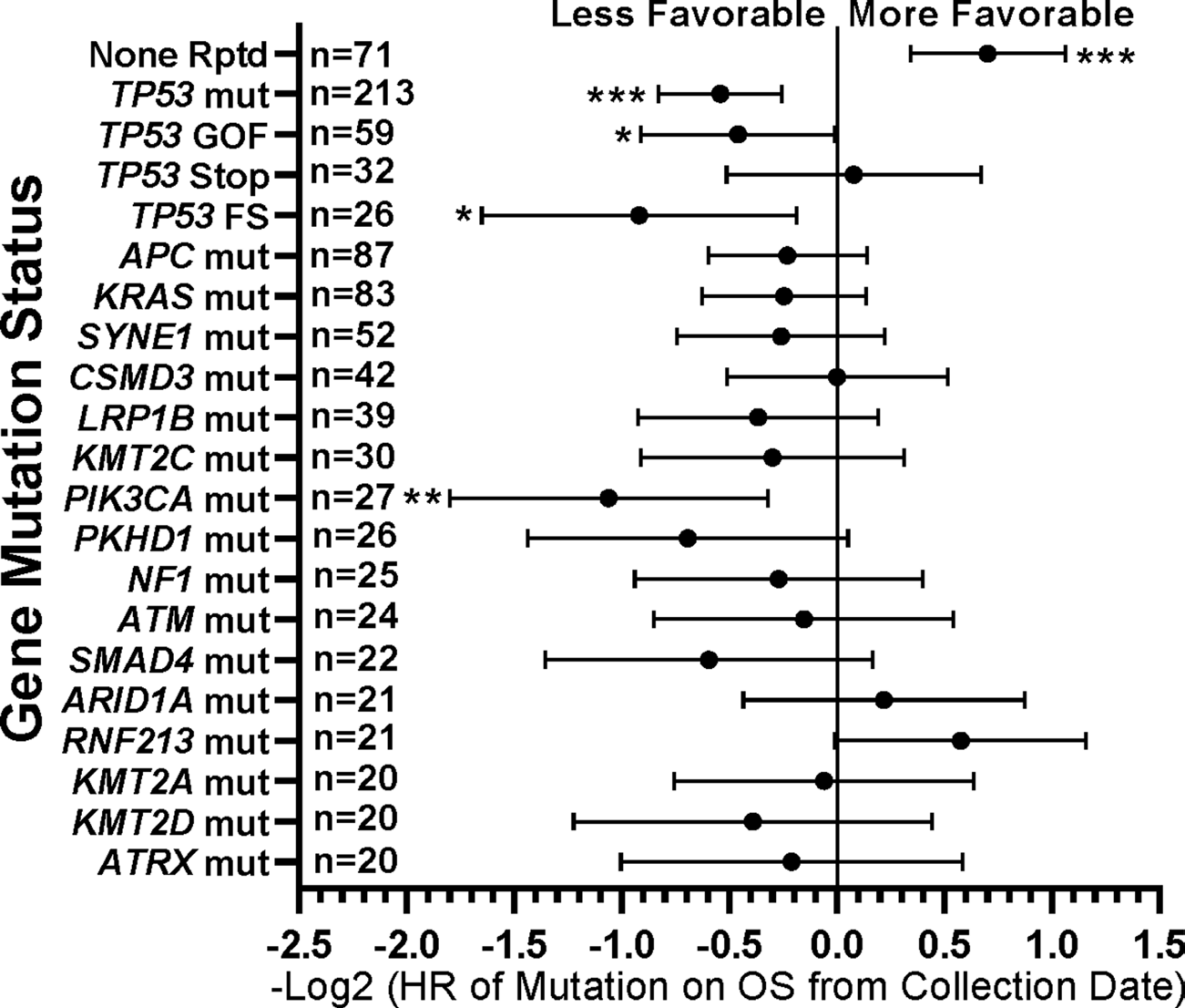
Summary of impact of individual gene mutations on survival. *TP53* gain-of-function (GOF) and frameshift (FS) mutations and *PIK3CA* mutations were associated with worse survival, while tumors with no mutations demonstrated more favorable survival. HRs are –Log2 transformed so that a HR of 2 equals 1.0 and a HR of 0.5 equals -1.0. ^****^ *p*-value < 0.0001; ^***^ *p*-value < 0.001; ^**^ *p*-value < 0.01, ^*^ *p*-value < 0.05. Since response to ICB was not interrogated in this relationships, all 554 patients were included in this analysis regardless of treatment status.

**Figure 8:**
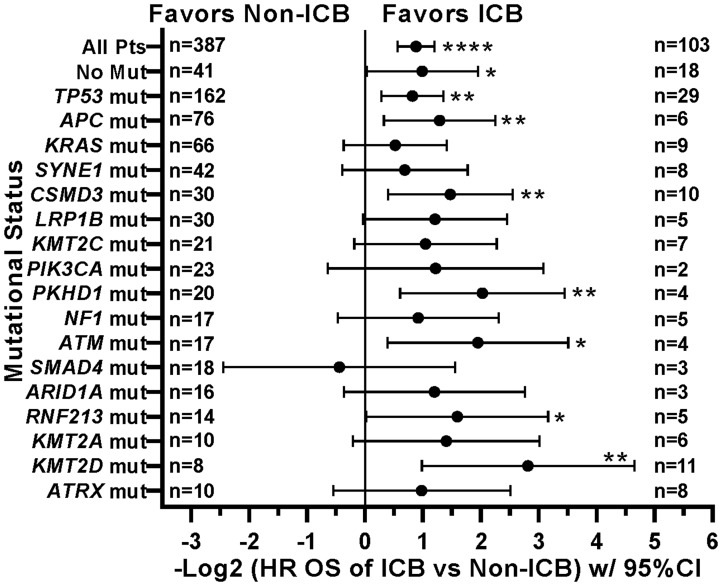
Effect of individual mutations and ICB treatment on survival. Patients with tumors with mutations in *TP53*, *APC*, *CSMD3*, *PKHD1*, *ATM*, *RNF213*, or *KMT2D* had improved overall survival when treated with ICB. HRs are –Log2 transformed so that a HR of 2 equals 1.0 and a HR of 0.5 equals -1.0. ^****^ *p*-value < 0.0001; ^***^ *p*-value < 0.001; ^**^ *p*-value < 0.01, ^*^ *p*-value < 0.05. Since response to ICB was interrogated in these relationships, only the 490 treated patients were included in this analysis.

**Figure 9:**
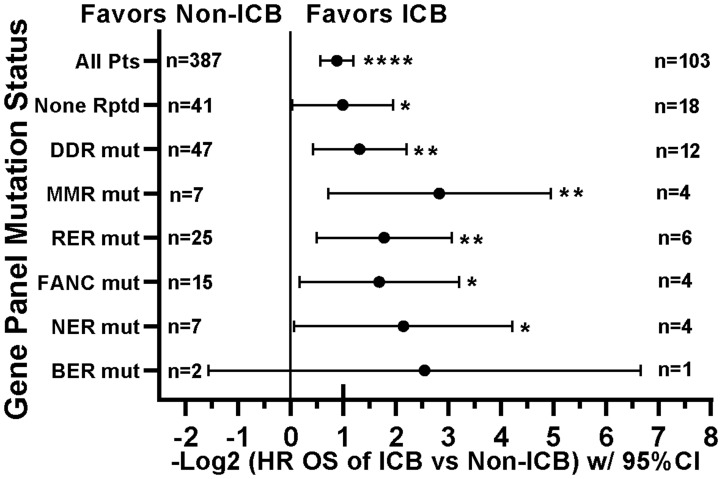
Tumors with mutations in DNA damage repair genes demonstrate improved survival with ICB versus non-ICB therapy. Although limited sample sizes prevent strong inferences, these patients show consistent response to ICB. The None Rptd (None Reported) group includes patients with no reported mutations in any of the panel (including DNA repair) genes. DDR (mutation in any DNA damage repair gene) group includes MMR, FA, BER, NER, RER, or CHEK. MMR (mutation in any mismatch repair gene) group includes MLH1, MSH2, MSH6, PMS1, or PMS2; RER (mutation in any recombination repair gene) group includes ATM, ATR, XRCC2, RAD50, WRN, PARP1, NBN, or MRE11A; FANC (mutation in any Fanconi anemia pathway gene) group includes FANCA, FANCC, FANCD2, FANCF, FANCG, PALB2, or BRIP1; NER (mutation in any nucleotide excision repair gene) group includes ERCC2, ERCC4, ERCC5, XPC, or XPA; BER (mutation in any base excision repair gene) group includes SMUG1 or MUTYH; CHEK (mutation in any checkpoint kinase gene) group includes CHEK1 or CHEK2. HRs are –Log2 transformed so that a HR of 2 equals 1.0 and a HR of 0.5 equals -1.0. ^****^ *p*-value < 0.0001; ^***^ *p*-value < 0.001; ^**^ *p*-value < 0.01, ^*^ *p*-value < 0.05, ^#^ *p*-value < 0.10. Since response to ICB was interrogated in these relationships, only the 490 treated patients were included in this analysis. HRs for groups with small numbers of patients are provided for completeness only.

**Figure 10:**
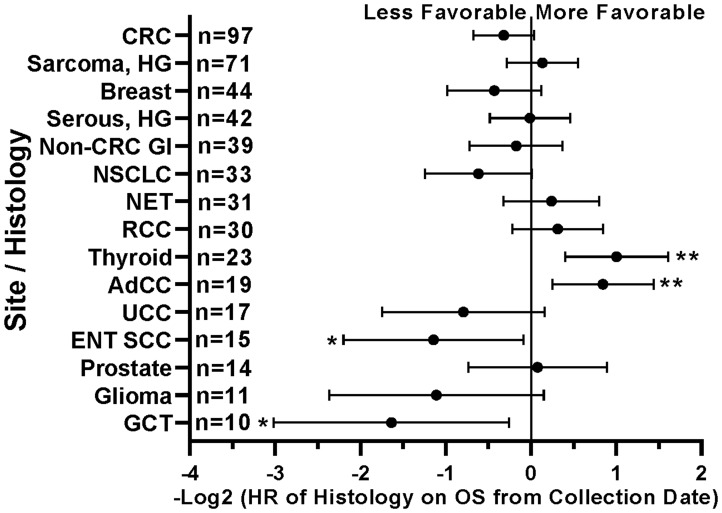
Impact of tumor histology/type on patient survival. Patients with head and neck squamous cell carcinomas (ENT SCC) and germ cell tumors (GCT) had less favorable survival. Patients with non-medullary thyroid carcinomas and adenoid cystic carcinomas (AdCC) demonstrated longer survival. HRs are –Log2 transformed so that a HR of 2 equals 1.0 and a HR of 0.5 equals -1.0. ^****^ *p*-value < 0.0001; ^***^ *p*-value < 0.001; ^**^ *p*-value < 0.01, ^*^ *p*-value < 0.05. Since response to ICB was not interrogated in these relationships, all 554 patients were included in this analysis regardless of treatment status.

**Figure 11:**
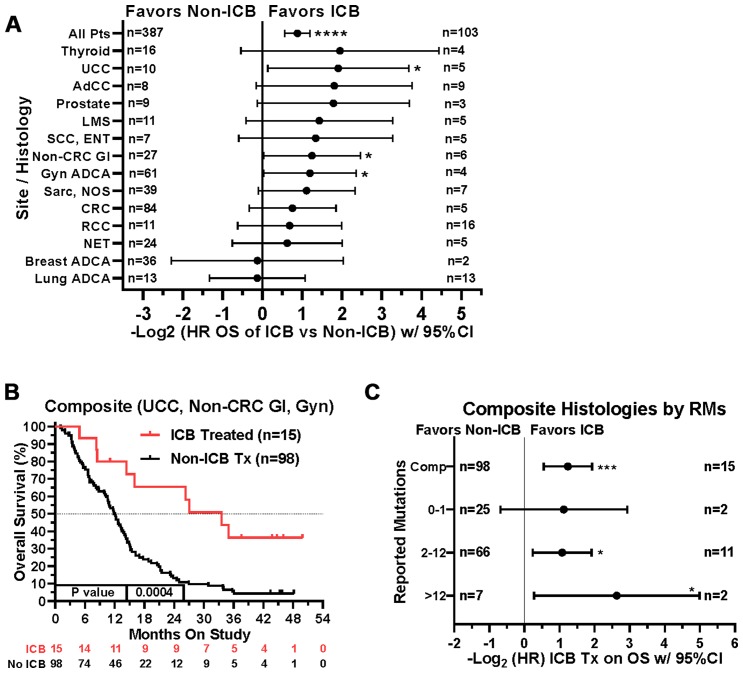
Impact of tumor type/site and ICB treatment on survival. (**A**) Patients with gynecologic adenocarcinomas, non-colorectal GI cancers, and urothelial carcinoma had improved overall survival when treated with ICB. Note that patient numbers are relatively small in the ICB treatment groups for most of the tumor types. Since response to ICB was interrogated in these relationships, only the 490 treated patients were included in this analysis. (**B**) If aggregated together into a composite clinical group (urothelial carcinomas, non-colorectal gastrointestinal carcinomas, and gynecologic carcinomas), the composite benefit of ICB therapy versus a non-ICB therapy (HR = 0.424, 95%CI [0.263 to 0.684], *n* = 15 *vs n* = 98, *p* = 0.0004) may be enriched by isolating on these tumor types. (**C**) A tree plot of hazard ratios of ICB treatment within this composite group of tumors is shown and stratified by number of reported mutations. HRs are –Log2 transformed so that a HR of 2 equals 1.0 and a HR of 0.5 equals -1.0. ^****^ *p*-value < 0.0001; ^***^ *p*-value < 0.001; ^**^ *p*-value < 0.01, ^*^ *p*-value < 0.05.

When comparing survival of the 490 treated patients in this study, mutations in a few other genes, specifically *APC*, *LRP1B*, and *PIK3CA*, were also associated with worse survival in all protocol patients by univariate analysis (Table 3). However, if comparing all patients irrespective of treatment, only tumors with any *TP53* or *PIK3CA* mutation demonstrated relatively worse survival (Figure 7). There were insufficient numbers of ICB treated patients with these tumor mutations to perform sub-analyses of ICB versus no ICB treatment. However, tumors with mutations in *TP53*, *APC*, *CSMD3*, *PKHD1*, *ATM*, *RNF213*, and *KMT2D* demonstrated improved overall survival when treated with ICB versus no ICB treatment (Figure 8) (*N* = 490). In addition, tumors with mutations in DNA damage repair genes demonstrated improved survival with ICB versus to non-ICB therapy (HR = 0.402 95%CI [0.217 to 0.746], *n* = 12 vs *n* = 47, *p* = 0.0039) (Figure 9). Interestingly, the improvement in hazard ratio of patients without a tumor mutant DR gene when treated on ICB was less than that seen in patients with tumors with mutated DR genes when treated with ICB was (HR = 0.564 95%CI [0.446 to 0.712], *n =* 91 vs *n =* 340, p<0.0001), though still highly significant. When comparing survival of the 490 treated patients in this study, univariate analyses showed that specific cancer types, such as colorectal adenocarcinoma, high grade ovarian/peritoneal serous carcinoma, and non-colorectal GI carcinoma were associated with a poorer survival (Table 3), whereas non-medullary thyroid carcinoma and adenoid cystic carcinoma were associated with improved survival in protocol patients (Table 3). However, when comparing all patients irrespective of treatment, only squamous cell carcinoma of the head and neck and germ cell tumors demonstrated significantly worse relative survival, whereas non-medullary thyroid carcinoma and adenoid cystic carcinoma again had relatively favorable survival (Figure 10). There were insufficient ICB-treated patients to examine impact of ICB in each of these cancer types, but gynecologic, non-colorectal GI, and urothelial carcinomas demonstrated improved overall survival when treated with ICB compared to non-ICB therapy on study (Figure 11A). Aggregating these three tumor types together into a composite clinical group highlights the benefit of ICB therapy versus a non-ICB therapy (HR = 0.424, 95%CI [0.263 to 0.684], *n =* 15 vs *n =* 98, *p =* 0.0004), demonstrating the ability to enrich for ICB response (Figure 11B). Breakdown of this composite group by TMB also showed variation among TMB groups (Figure 11C). Interestingly, the data indicate significant responses to ICB in the middle and high TMB groups.

Our initial simplified multivariate analysis demonstrated that both ICB treatment and tumor mutation burden were associated with overall survival. A more complex multivariate analysis that included treatment type, cancer type, specific gene mutations, and TMB, treatment with ICB remained strongly associated with better overall survival (Table 3). A few other factors, such as presence of specific tumor histologies (adenoid cystic carcinoma and non-medullary thyroid carcinoma; favorable), and presence of *LRP1B* mutation (unfavorable), retained their respective associations with survival (Table 3). Notably, in the more comprehensive multivariate analysis, TMB by itself was not associated with survival after taking into account a number of other histologic and genetic mutational variables. These results therefore suggest that the relationship between high TMB and beneficial response to ICB therapy may be more complex than previously thought. Tumor type and mutations in individual genes may be modifiers of the impact high TMB has on ICB treatment response.

## DISCUSSION

In patients with stage III/IV advanced solid malignancies refractory to standard treatment, our data argue for inclusion of ICB treatment as a therapeutic consideration. We found worsening survival outcomes with increasing TMB which may be reversed by treatment with ICB. Our data suggest that increasing TMB may actually be associated with improved survival in the ICB treated subgroup of patients. This complex relationship between TMB, ICB treatment, and survival highlights the importance of tumor mutational profiling in this patient population. Such molecular profiling not only identifies potential targets for matched targeted therapy [2], it can identify patients with tumors with higher TMB who may be more optimal candidates for ICB. Failure to recognize this potential positive impact of ICB on survival in this difficult to treat patient population could therefore represent an important lost opportunity.

The limitations of this study include the fact that the treatments for this patient population were not randomized. We cannot exclude the potential for selection bias in choosing to give ICB therapy to patients who may already be more likely to demonstrate prolonged survival. However, this report does suggest that ICB-based trials may be more likely to provide benefit in a wider spectrum of cancer types in patients who have advanced disease.

Prior evaluations of the prognostic value of TMB have mainly focused upon a “hypermutated” group (>8 to 17 /Mb), showing high TMB as a good prognostic biomarker [20]. This hypermutated group typically consists only of 5–20% of a given cancer patient population. The majority of patients with solid tumors (80–95%) fall within the TMB “low” group (<8 to 17 /MB). Within this larger group of TMB low patients, increasing TMB may reflect increasing tumor complexity and heterogeneity leading to treatment refractoriness to conventional therapy and unfavorable outcomes. In contrast, tumors with very low TMB (0–1 /Mb) may have less intra-tumoral heterogeneity, and patients may respond more favorably to conventional therapy. The NGS panel in our study was 1.745 Mb, somewhat larger than most commercially available NGS panels. Our data argue for the utility of a larger NGS panel to identify TMB at a more granular level, especially on the lower end of the TMB spectrum. In fact, a number of studies have emphasized the utility of larger panels in this range in order to obtain sufficient sensitivity, especially when interrogating tumors with less than 10 somatic mutations per Mb [21–23].

The optimal approach to identify cancer patients who are most likely to benefit from immunotherapy remains controversial. PD-L1 Immunohistochemical assessment in solids tumors has demonstrated a moderate association with response to ICB (PD-L1 positive: 48% vs PD-L1 negative: 15%) [24]. However, in renal cell carcinoma [24], PD-L1 estimation has not been associated with response to ICB. PD-L1 expression has only modest overlap with TMB high tumors, capturing approximately 45% of tumors with higher TMB [25]. In fact, PD-L1 expression by immunohistochemistry has been shown to be somewhat independent of TMB in non-small cell lung carcinoma [26]. Mismatch repair deficiency and high levels of microsatellite instability are now approved indications for ICB therapy [27]. Mismatch repair and microsatellite instability testing have been limited largely to gastrointestinal and endometrial carcinomas due to low identification rates in other tumor types [27]. Microsatellite instability testing captures only 10–30% of TMB high tumors [25, 28]. In colorectal carcinoma, the overlap between mismatch repair deficiency and TMB is strong, but this is not true in endometrial, ovarian, cervical and neuroendocrine carcinomas [25]. Given that PD-L1 immunohistochemistry, mismatch repair immunohistochemistry, and microsatellite instability are relatively simple clinical laboratory assays that are widely available, a simplified approach might include utilization of these tests first. If they are negative, then the more complex assessment of TMB via a large scale NGS panel could be pursued.

A better understanding of the impact of individual gene alterations on patient outcomes with ICB treatment is emerging. For example, in *KRAS*-mutant lung adenocarcinoma, *STK11/LKB1* mutation is associated with resistance to ICB treatment [29]. Co-mutations of TP53 and KRAS in lung adenocarcinoma are reported to correlate with improved efficacy of ICB therapy [30]. Mutations of *LRP1B* are associated with increased TMB and survival in melanoma and non-small cell lung carcinoma patients given immunotherapy [31]. Loss of tumor PTEN protein expression in melanoma is associated with worse ICB outcomes and reduced tumor infiltration by CD8+ T cells [32]. *TP53* mutation was the most common molecular alteration in our patient population and was associated with worse survival, similar to its known association with unfavorable outcome in solid and hematologic malignancies [33–36]. Our data demonstrate that while TP53 mutations are associated with unfavorable survival, the effect of ICB therapy is not diminished by the presence of this gene mutation. This is concordant with previous data showing PD-L1 expression may be increased in TP53 and KRAS mutated non-small cell lung carcinomas [30]. Our data also suggest that tumor mutations in a broad array of DNA repair genes is associated with better response to ICB therapy. Such DNA repair pathways include mismatch repair (MMR), base excision repair (BER), nucleotide excision repair (NER), recombination repair (RER), and Fanconi anemia (FA).

Finally, in the expanded multivariate analysis, treatment with ICB retained its association with improved survival, independent of all other variables in the model. The loss of significance of TMB in the multivariate analysis may be due to high correlation of TMB with cancer type and/or specific mutational profiles [28, 37]. Our findings may corroborate the observation that the ability of TMB to predict ICB benefit is dependent upon histologic type of cancer [38]. If true, this argues for the use of histology specific TMB cutoffs as well as modifications based on mutational profile in future clinical practice.

Taken together, although our initial multivariate analysis demonstrated that both ICB treatment and tumor mutation burden were *associated with overall* survival, a more complex multivariate analysis that included treatment type, cancer type, specific gene mutations, and TMB, demonstrated only treatment with ICB remained strongly associated with better overall survival (Table 3). A few other factors, such as presence of specific tumor histologies (adenoid cystic carcinoma and non-medullary thyroid carcinoma; favorable), and presence of *LRP1B* mutation (unfavorable), retained their respective associations with survival (Table 3). Notably, in the more comprehensive multivariate analysis, TMB was not associated with survival after taking into account histologic and genetic mutational variables. These results therefore suggest that the relationship between high TMB and beneficial response to ICB therapy may be more complex than previously thought and that tumor type and mutations in individual genes should also be considered when constructing clinical models predictive of response to ICB.

## MATERIALS AND METHODS

### Patient population

As described previously (*2*), patients were enrolled prospectively into an IRB-approved institutional protocol, PA14–0099, between May 7, 2014, and October 5, 2015. Written informed consent from all patients participating in this trial was obtained prior to enrollment. Key enrollment criteria included: 1) any adult patient with pathologic documentation of a single solid malignancy; 2) completion of frontline and any standard treatments that extended life by at least 3 months; 3) Eastern Cooperative Oncology Group performance status of 0 or 1; 4) no active brain metastases; and 5) previous tumor testing using a smaller sequencing panel and that showed no clinically actionable mutations or the patient had progressed on a matched therapy targeting a previously identified actionable finding. Electronic medical records were reviewed for specific treatment on study, including any type of ICB treatment. ICB therapy on study was defined as treatment with any CTLA-4 (ipilimumab, tremelimumab), PD-1 (nivolumab, pembrolizumab, spartalizumab, cemiplimab), and/or PD-L1 (atezolizumab, durvalumab) monoclonal antibody after the trial consent date.

### Molecular testing

Molecular testing was performed on formalin-fixed, paraffin-embedded (FFPE) tissue of patient tumors using a NGS panel that covers the entire coding regions of 409 cancer-related genes (15,992 amplicons; 1.745 Mb) as previously detailed [39]. Subtraction of germline single nucleotide polymorphisms (SNPs) was performed for each patient using a paired normal control specimen derived from either peripheral blood mononuclear cells or normal FFPE tissue from the same patient. For each patient tumor, TMB was calculated as the sum of reported somatic mutations (including SNV and indels), and not counting intronic or silent mutations. TMB can also be expressed as mutations /Mb by dividing the number of reported mutations by the panel footprint of 1.745 Mb. Supplementary Table 3 summarizes the number of reported tumor mutations and the corresponding TMB (mutations/MB). TP53 somatic mutations were the most common in this patient population, and they were further sub-categorized as gain-of-function, frameshift, and/or stop (nonsense). Gain-of-function mutations may gain novel functions through protein–protein interactions with the transcription factors NF-Y, VDR, PML, Sp1, and Ets2 [40].

### Mismatch repair and PD-L1 immunohistochemistry and microsatellite instability analyses

Mismatch repair protein immunohistochemistry was performed according to methodology previously described [41]. Briefly, immunohistochemistry of mismatch repair proteins was performed using standard techniques for MLH1 (G168–15 1:25; BD Biosciences Pharmingen), MSH2 (FE11, 1:100; Calbiochem), MSH6 (44, 1:300; BD Biosciences Pharmingen), and PMS2 (Alb-4, 1:125; BD Biosciences Pharmingen). Immunohistochemistry was scored as mismatch repair protein intact or deficient using light microscopic examination. Complete absence of mismatch repair protein expression was required in order for a case to be designated as mismatch repair deficient. Stromal cells served as an internal positive control. For PD-L1 immunohistochemistry, staining and analysis were performed using clones 28–8 and 22C3 (Dako). Any positive staining in tumor cells observed by light microscopy was considered PD-L1 positive. PCR-based microsatellite instability analysis was performed using 6 National Cancer Institute (Bethesda, MD) recommended microsatellites with the addition of TGFβR2 as previously described [42].

### Statistical analyses

Survival was calculated from date of study consent to date of death or last known encounter within the medical record. GraphPad Prism 8.1.2 software (La Jolla, CA) was used to plot histograms and Kaplan-Meier survival curves and to calculate *p*-values for Mantel-Haenszel hazard ratios (HRs) with 95% confidence intervals and log-rank tests for trend. Recursive partitioning for classification and tree methods [43] were used to determine the optimal cut points for reported mutations in the ICB treated group. With the determined cut points above, Kaplan-Meier curves were plotted for ICB-treated, non-ICB treated, and all patients on study, and the *p*-values from the log-rank tests were provided. Cox proportional hazard models were utilized for univariate *and multivariate analyses* on the time-to-event endpoint of overall survival. For multivariate analysis, variables with *p*-values less than 0.2 from the univariate model were considered for model fitting and stepwise model selection, and the variables with *p*-values less than 0.05 were considered statistically significant in the final model. To determine the best functional form of the continuous covariates in the model with ICB and tumor mutation burden, the martingale residuals were plotted for the variable tumor mutation burden along with locally weighted scatterplot smoothing after a Cox model with other variable (s) was fitted. The smoothed curve was not linear, suggesting that transformation of tumor mutation burden was necessary. Since the distribution of tumor mutation burden was positively skewed, and there were many zero and small values for tumor mutation burden, the square root transformation of tumor mutation burden was utilized. Correction for multiple comparisons was not performed due to the retrospective exploratory nature of this analysis. All tests were two-sided. P-values less than 0.05 were considered statistically significant. In addition to GraphPad Prism, the analyses were performed using R version 3.5.3 (2019–03-11) and SAS 9.4 (SAS, Cary, NC).

### Ethics approval

This study protocol (PA14–0099) was approved by the MD Anderson Cancer Center Institutional Review Board. Written consent from all patients participating in this trial was obtained prior to enrollment.

## SUPPLEMENTARY MATERIALS






