# Supplementary material for: Identification of biomarkers of immune checkpoint blockade efficacy in recurrent or refractory solid tumor malignancies
Source: Oncotarget. 2020 Feb 11;11(6):600–18. doi: 10.18632/oncotarget.27466 (PMC7021232; doi:10.18632/oncotarget.27466)
Supplement: Supplementary file 1 [file oncotarget-11-600-s001.pdf]

Identification of biomarkers of immune checkpoint blockade efficacy in recurrent or refractory solid tumor malignancies

SUPPLEMENTARY MATERIALS

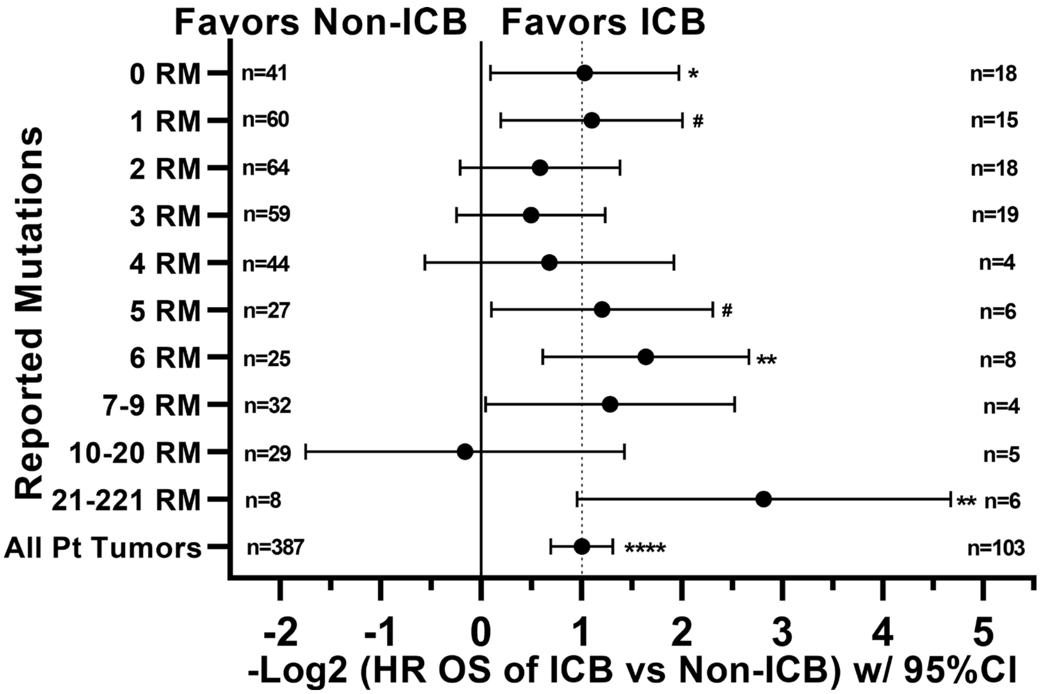

Supplementary Figure 1: Patients with high TMB benefit most from ICB versus non-ICB treatment. HRs are –Log2 transformed so that a HR of 2 equals 1.0 and a HR of 0.5 equals -1.0. ****p-value < 0.0001; ***p-value < 0.001; **p-value < 0.01, *p-value < 0.05, #p-value < 0.10. In the above chart, each RM (reported mutations) is equivalent to 0.573/Mb worth of tumor mutation burden (TMB). For example, two (2) RMs (reported mutations) is equivalent to 1.146/Mb of TMB, 10 RMs (reported mutations) is equivalent to 5.73/Mb) of TMB, 100 RMs (reported mutations) is equivalent to 57.3/Mb) of TMB, et cetera. Since response to ICB was interrogated in these relationships, only the 490 treated patients were included in this analysis.

Supplementary Table 1: Patient characteristics. See Supplementary Table 1

Supplementary Table 2: Patient characteristics expressed as percentages (%). See Supplementary Table 2

Supplementary Table 3: Reported mutation to tumor mutational burden (TMB) conversion chart

Reported Mutations	Footprint	TMB (mutations /Mb)
1 RM	1.745 MB	0.573
2 RM	1.745 MB	1.146
3 RM	1.745 MB	1.719
4 RM	1.745 MB	2.292
5 RM	1.745 MB	2.865
6 RM	1.745 MB	3.438
7 RM	1.745 MB	4.011
8 RM	1.745 MB	4.585
9 RM	1.745 MB	5.158
10 RM	1.745 MB	5.731
11 RM	1.745 MB	6.304
12 RM	1.745 MB	6.877
13 RM	1.745 MB	7.450
14 RM	1.745 MB	8.023
15 RM	1.745 MB	8.596
16 RM	1.745 MB	9.169
17 RM	1.745 MB	9.742
18 RM	1.745 MB	10.315
19 RM	1.745 MB	10.888
20 RM	1.745 MB	11.461
21 RM	1.745 MB	12.034
22 RM	1.745 MB	12.607
25 RM	1.745 MB	14.327
30 RM	1.745 MB	17.192
40 RM	1.745 MB	22.923
50 RM	1.745 MB	28.653
100 RM	1.745 MB	57.307
200 RM	1.745 MB	114.613
500 RM	1.745 MB	286.533
1000 RM	1.745 MB	573.066
