# Supplementary material for: Identification of biomarkers of immune checkpoint blockade efficacy in recurrent or refractory solid tumor malignancies
Source: Oncotarget. 2020 Feb 11;11(6):600–18. doi: 10.18632/oncotarget.27466 (PMC7021232; doi:10.18632/oncotarget.27466)
Supplement: Supplementary file 2 [file oncotarget-11-600-s002.docx]

**Supplementary Table 1: Patient Characteristics**

|  |  |  | **Treatment** | | **RMs** |  |  | **Mutated Gene** | | | |  |  |  |  |  |  |  |  |  |  |  |  |  |
| --- | --- | --- | --- | --- | --- | --- | --- | --- | --- | --- | --- | --- | --- | --- | --- | --- | --- | --- | --- | --- | --- | --- | --- | --- |
|  | **# Pts** | **% Pts** | **ICB** | **No ICB** | **0 to 1** | **2 to 12** | **>12** | **TP53** | **APC** | **KRAS** | **SYNE1** | **CSMD3** | **LRP1B** | **MLL3** | **PIK3CA** | **PKHD1** | **NF1** | **ATM** | **SMAD4** | **ARID1A** | **RNF213** | **MLL** | **MLL2** | **ATRX** |
| **Sex** |  |  |  |  |  |  |  |  |  |  |  |  |  |  |  |  |  |  |  |  |  |  |  |  |
| Female | 256 | 52.2 | 46 | 210 | 30 | 170 | 13 | 112 | 37 | 45 | 24 | 17 | 16 | 12 | 17 | 9 | 11 | 9 | 7 | 11 | 7 | 8 | 8 | 10 |
| Male | 234 | 47.8 | 57 | 177 | 29 | 158 | 17 | 79 | 45 | 30 | 26 | 23 | 19 | 16 | 8 | 15 | 11 | 12 | 14 | 8 | 12 | 8 | 11 | 8 |
| **Histology** |  |  |  |  |  |  |  |  |  |  |  |  |  |  |  |  |  |  |  |  |  |  |  |  |
| Colorectal ADCA | 87 | 17.8 | 5 | 82 | 2 | 80 | 5 | 59 | 65 | 48 | 22 | 10 | 5 | 5 | 8 | 2 | 4 | 6 | 12 | 2 | 2 | 2 | 1 | 2 |
| Sarcoma, HG | 64 | 13.1 | 13 | 51 | 26 | 36 | 2 | 15 | 1 | 0 | 1 | 6 | 7 | 0 | 1 | 4 | 3 | 1 | 0 | 0 | 1 | 2 | 1 | 6 |
| Breast ADCA | 38 | 7.8 | 2 | 36 | 7 | 30 | 1 | 19 | 1 | 1 | 7 | 2 | 1 | 3 | 7 | 1 | 0 | 1 | 0 | 1 | 0 | 1 | 0 | 1 |
| Serous, HG | 37 | 7.6 | 0 | 37 | 10 | 27 | 0 | 30 | 0 | 0 | 0 | 1 | 1 | 3 | 0 | 1 | 0 | 1 | 0 | 2 | 1 | 1 | 0 | 1 |
| Non CRC GI | 33 | 6.7 | 6 | 27 | 10 | 21 | 2 | 13 | 4 | 8 | 3 | 2 | 4 | 2 | 2 | 3 | 1 | 0 | 6 | 0 | 3 | 0 | 1 | 0 |
| NSCLC | 29 | 5.9 | 15 | 14 | 8 | 18 | 3 | 7 | 1 | 9 | 2 | 2 | 4 | 1 | 1 | 0 | 3 | 0 | 0 | 1 | 2 | 1 | 3 | 1 |
| Renal Cell ADCA | 28 | 5.7 | 16 | 12 | 8 | 20 | 0 | 6 | 0 | 1 | 1 | 2 | 1 | 1 | 0 | 0 | 0 | 0 | 0 | 1 | 1 | 0 | 1 | 1 |
| Thyroid carcinoma | 20 | 4.1 | 4 | 16 | 14 | 6 | 0 | 2 | 0 | 0 | 0 | 0 | 0 | 1 | 0 | 0 | 0 | 2 | 0 | 1 | 0 | 0 | 1 | 0 |
| Adenoid Cystic | 17 | 3.5 | 9 | 8 | 12 | 5 | 0 | 0 | 0 | 0 | 1 | 1 | 0 | 1 | 0 | 0 | 0 | 0 | 0 | 0 | 0 | 0 | 0 | 1 |
| Urothelial ADCA | 15 | 3.1 | 5 | 10 | 0 | 11 | 4 | 7 | 4 | 1 | 1 | 6 | 0 | 2 | 1 | 1 | 2 | 4 | 0 | 3 | 0 | 4 | 4 | 1 |
| Head & Neck SCC | 12 | 2.4 | 4 | 8 | 1 | 10 | 1 | 3 | 0 | 0 | 0 | 2 | 0 | 1 | 1 | 1 | 0 | 1 | 0 | 0 | 0 | 2 | 0 | 0 |
| Prostate ADCA | 12 | 2.4 | 3 | 9 | 4 | 7 | 1 | 5 | 0 | 0 | 1 | 1 | 1 | 2 | 0 | 1 | 0 | 1 | 0 | 1 | 1 | 0 | 0 | 0 |
| Glioma, HG | 9 | 1.8 | 0 | 9 | 2 | 5 | 2 | 4 | 1 | 0 | 1 | 0 | 1 | 1 | 1 | 1 | 2 | 0 | 0 | 2 | 1 | 0 | 1 | 1 |
| Endometrioid ADCA | 7 | 1.4 | 0 | 7 | 0 | 6 | 1 | 3 | 1 | 2 | 0 | 0 | 1 | 0 | 1 | 2 | 0 | 0 | 0 | 3 | 0 | 0 | 0 | 0 |
| Germ Cell Tumors | 5 | 1.0 | 0 | 5 | 0 | 5 | 0 | 0 | 0 | 0 | 0 | 0 | 1 | 1 | 0 | 0 | 0 | 0 | 0 | 0 | 0 | 0 | 0 | 0 |
| Other | 77 | 15.7 | 21 | 56 | 30 | 39 | 8 | 18 | 4 | 5 | 10 | 5 | 8 | 4 | 2 | 7 | 7 | 4 | 3 | 2 | 7 | 3 | 6 | 3 |
| Total | 490 | 100.0 | 103 | 387 | 134 | 326 | 30 | 191 | 82 | 75 | 50 | 40 | 35 | 28 | 25 | 24 | 22 | 21 | 21 | 19 | 19 | 16 | 19 | 18 |
| **ICB post OSD** |  |  |  |  |  |  |  |  |  |  |  |  |  |  |  |  |  |  |  |  |  |  |  |  |
| Any ICB post-OSD | 103 | 21.0 | 103 | 0 | 33 | 61 | 9 | 29 | 6 | 9 | 8 | 10 | 5 | 7 | 2 | 4 | 5 | 4 | 3 | 3 | 5 | 6 | 11 | 8 |
| anti-PD-1 post-OSD | 75 | 15.3 | 75 | 0 | 23 | 46 | 6 | 22 | 4 | 5 | 6 | 6 | 3 | 5 | 1 | 3 | 2 | 3 | 0 | 1 | 4 | 4 | 10 | 6 |
| anti-PD-L1 post-OSD | 22 | 4.5 | 22 | 0 | 5 | 15 | 2 | 10 | 2 | 5 | 1 | 4 | 1 | 3 | 1 | 1 | 3 | 1 | 3 | 2 | 1 | 1 | 1 | 2 |
| anti-CTLA4 post-OSD | 28 | 5.7 | 28 | 0 | 14 | 12 | 2 | 3 | 1 | 2 | 2 | 1 | 1 | 0 | 1 | 0 | 1 | 1 | 0 | 0 | 0 | 2 | 3 | 0 |
| Any Non-ICB Tx post-OSD | 387 | 79.0 | 0 | 387 | 101 | 267 | 21 | 161 | 74 | 66 | 41 | 30 | 30 | 20 | 23 | 20 | 17 | 17 | 18 | 16 | 14 | 10 | 8 | 10 |
| **Age at OSD** |  |  |  |  |  |  |  |  |  |  |  |  |  |  |  |  |  |  |  |  |  |  |  |  |
| <20 | 6 | 1.2 | 0 | 6 | 3 | 2 | 1 | 3 | 1 | 0 | 1 | 0 | 0 | 1 | 0 | 1 | 0 | 0 | 0 | 1 | 1 | 1 | 1 | 1 |
| 20s | 21 | 4.3 | 3 | 18 | 6 | 15 | 0 | 3 | 0 | 0 | 2 | 2 | 1 | 0 | 0 | 1 | 2 | 0 | 0 | 0 | 0 | 0 | 0 | 0 |
| 30s | 37 | 7.6 | 6 | 31 | 20 | 15 | 2 | 8 | 2 | 2 | 0 | 3 | 3 | 2 | 2 | 0 | 1 | 1 | 0 | 0 | 1 | 2 | 1 | 0 |
| 40s | 86 | 17.6 | 20 | 66 | 21 | 60 | 5 | 36 | 23 | 16 | 11 | 6 | 5 | 1 | 4 | 4 | 3 | 4 | 2 | 2 | 3 | 3 | 3 | 4 |
| 50s | 142 | 29.0 | 36 | 105 | 36 | 98 | 7 | 58 | 23 | 26 | 21 | 11 | 9 | 8 | 7 | 4 | 7 | 3 | 9 | 5 | 6 | 4 | 4 | 7 |
| 60s | 131 | 26.7 | 26 | 104 | 28 | 91 | 11 | 60 | 23 | 23 | 10 | 9 | 9 | 12 | 6 | 5 | 7 | 9 | 8 | 7 | 5 | 4 | 8 | 2 |
| 70s | 63 | 12.9 | 11 | 52 | 18 | 41 | 4 | 22 | 8 | 7 | 4 | 9 | 8 | 3 | 6 | 9 | 2 | 2 | 2 | 3 | 3 | 2 | 2 | 4 |
| >80 | 6 | 1.2 | 1 | 5 | 2 | 4 | 0 | 0 | 0 | 1 | 0 | 0 | 0 | 0 | 0 | 0 | 0 | 2 | 0 | 1 | 0 | 0 | 0 | 0 |
| **All Treated** | 490 | 100.0 | 103 | 387 | 134 | 326 | 30 | 190 | 80 | 75 | 49 | 40 | 35 | 27 | 25 | 24 | 22 | 21 | 21 | 19 | 19 | 16 | 19 | 18 |

Abbreviations: ADCA- Adenocarcinoma; GI - Gastrointestinal; HG - High grade; CRC - Colorectal; OSD - On Study Date; SCC - Squamous Cell Carcinoma.
