# Supplementary material for: Identification of biomarkers of immune checkpoint blockade efficacy in recurrent or refractory solid tumor malignancies
Source: Oncotarget. 2020 Feb 11;11(6):600–18. doi: 10.18632/oncotarget.27466 (PMC7021232; doi:10.18632/oncotarget.27466)
Supplement: Supplementary file 3 [file oncotarget-11-600-s003.docx]

**Supplementary Table 2: Patient Characteristics Expressed as Percentages (%)**

|  |  |  | **Treatment** | | **RMs** |  |  | **Mutated Gene** | | | |  |  |  |  |  |  |  |  |  |  |  |  |  |
| --- | --- | --- | --- | --- | --- | --- | --- | --- | --- | --- | --- | --- | --- | --- | --- | --- | --- | --- | --- | --- | --- | --- | --- | --- |
|  | **# Pts** | **% Pts** | **% ICB** | **% No ICB** | **% 0 to 1** | **% 2 to 12** | **% >12** | **% TP53** | **% APC** | **% KRAS** | **% SYNE1** | **% CSMD3** | **% LRP1B** | **% MLL3** | **% PIK3CA** | **% PKHD1** | **% NF1** | **% ATM** | **% SMAD4** | **% ARID1A** | **% RNF213** | **% MLL** | **% MLL2** | **% ATRX** |
| **Sex** |  |  |  |  |  |  |  |  |  |  |  |  |  |  |  |  |  |  |  |  |  |  |  |  |
| Female | 256 | 52 | 18 | 82 | 12 | 66 | 5 | 44 | 14 | 18 | 9 | 7 | 6 | 5 | 7 | 4 | 4 | 4 | 3 | 4 | 3 | 3 | 3 | 4 |
| Male | 234 | 48 | 24 | 76 | 12 | 68 | 7 | 34 | 19 | 13 | 11 | 10 | 8 | 7 | 3 | 6 | 5 | 5 | 6 | 3 | 5 | 3 | 5 | 3 |
| **Histology** |  |  |  |  |  |  |  |  |  |  |  |  |  |  |  |  |  |  |  |  |  |  |  |  |
| Colorectal ADCA | 87 | 18 | 6 | 94 | 2 | 92 | 6 | 68 | 75 | 55 | 25 | 11 | 6 | 6 | 9 | 2 | 5 | 7 | 14 | 2 | 2 | 2 | 1 | 2 |
| Sarcoma, HG | 64 | 13 | 20 | 80 | 41 | 56 | 3 | 23 | 2 | 0 | 2 | 9 | 11 | 0 | 2 | 6 | 5 | 2 | 0 | 0 | 2 | 3 | 2 | 9 |
| Breast ADCA | 38 | 8 | 5 | 95 | 18 | 79 | 3 | 50 | 3 | 3 | 18 | 5 | 3 | 8 | 18 | 3 | 0 | 3 | 0 | 3 | 0 | 3 | 0 | 3 |
| Serous, HG | 37 | 8 | 0 | 100 | 27 | 73 | 0 | 81 | 0 | 0 | 0 | 3 | 3 | 8 | 0 | 3 | 0 | 3 | 0 | 5 | 3 | 3 | 0 | 3 |
| Non CRC GI | 33 | 7 | 18 | 82 | 30 | 64 | 6 | 39 | 12 | 24 | 9 | 6 | 12 | 6 | 6 | 9 | 3 | 0 | 18 | 0 | 9 | 0 | 3 | 0 |
| NSCLC | 29 | 6 | 52 | 48 | 28 | 62 | 10 | 24 | 3 | 31 | 7 | 7 | 14 | 3 | 3 | 0 | 10 | 0 | 0 | 3 | 7 | 3 | 10 | 3 |
| Renal Cell ADCA | 28 | 6 | 57 | 43 | 29 | 71 | 0 | 21 | 0 | 4 | 4 | 7 | 4 | 4 | 0 | 0 | 0 | 0 | 0 | 4 | 4 | 0 | 4 | 4 |
| Thyroid carcinoma | 20 | 4 | 20 | 80 | 70 | 30 | 0 | 10 | 0 | 0 | 0 | 0 | 0 | 5 | 0 | 0 | 0 | 10 | 0 | 5 | 0 | 0 | 5 | 0 |
| Adenoid Cystic | 17 | 3 | 53 | 47 | 71 | 29 | 0 | 0 | 0 | 0 | 6 | 6 | 0 | 6 | 0 | 0 | 0 | 0 | 0 | 0 | 0 | 0 | 0 | 6 |
| Urothelial ADCA | 15 | 3 | 33 | 67 | 0 | 73 | 27 | 47 | 27 | 7 | 7 | 40 | 0 | 13 | 7 | 7 | 13 | 27 | 0 | 20 | 0 | 27 | 27 | 7 |
| Head & Neck SCC | 12 | 2 | 33 | 67 | 8 | 83 | 8 | 25 | 0 | 0 | 0 | 17 | 0 | 8 | 8 | 8 | 0 | 8 | 0 | 0 | 0 | 17 | 0 | 0 |
| Prostate ADCA | 12 | 2 | 25 | 75 | 33 | 58 | 8 | 42 | 0 | 0 | 8 | 8 | 8 | 17 | 0 | 8 | 0 | 8 | 0 | 8 | 8 | 0 | 0 | 0 |
| Glioma, HG | 9 | 2 | 0 | 100 | 22 | 56 | 22 | 44 | 11 | 0 | 11 | 0 | 11 | 11 | 11 | 11 | 22 | 0 | 0 | 22 | 11 | 0 | 11 | 11 |
| Endometrioid ADCA | 7 | 1 | 0 | 100 | 0 | 86 | 14 | 43 | 14 | 29 | 0 | 0 | 14 | 0 | 14 | 29 | 0 | 0 | 0 | 43 | 0 | 0 | 0 | 0 |
| Germ Cell Tumors | 5 | 1 | 0 | 100 | 0 | 100 | 0 | 0 | 0 | 0 | 0 | 0 | 20 | 20 | 0 | 0 | 0 | 0 | 0 | 0 | 0 | 0 | 0 | 0 |
| Others | 77 | 16 | 27 | 73 | 39 | 51 | 10 | 23 | 5 | 6 | 13 | 6 | 10 | 5 | 3 | 9 | 9 | 5 | 4 | 3 | 9 | 4 | 8 | 4 |
| **ICB post OSD** |  |  |  |  |  |  |  |  |  |  |  |  |  |  |  |  |  |  |  |  |  |  |  |  |
| Any ICB post-OSD | 103 | 21 | 100 | 0 | 32 | 59 | 9 | 28 | 6 | 9 | 8 | 10 | 5 | 7 | 2 | 4 | 5 | 4 | 3 | 3 | 5 | 6 | 11 | 8 |
| anti-PD-1 post-OSD | 75 | 15 | 100 | 0 | 31 | 61 | 8 | 29 | 5 | 7 | 8 | 8 | 4 | 7 | 1 | 4 | 3 | 4 | 0 | 1 | 5 | 5 | 13 | 8 |
| anti-PD-L1 post-OSD | 22 | 4 | 100 | 0 | 23 | 68 | 9 | 45 | 9 | 23 | 5 | 18 | 5 | 14 | 5 | 5 | 14 | 5 | 14 | 9 | 5 | 5 | 5 | 9 |
| anti-CTLA4 post-OSD | 28 | 6 | 100 | 0 | 50 | 43 | 7 | 11 | 4 | 7 | 7 | 4 | 4 | 0 | 4 | 0 | 4 | 4 | 0 | 0 | 0 | 7 | 11 | 0 |
| Any Non-ICB Tx post-OSD | 387 | 79 | 0 | 100 | 26 | 69 | 5 | 42 | 19 | 17 | 11 | 8 | 8 | 5 | 6 | 5 | 4 | 4 | 5 | 4 | 4 | 3 | 2 | 3 |
| **Age at OSD** |  |  |  |  |  |  |  |  |  |  |  |  |  |  |  |  |  |  |  |  |  |  |  |  |
| <20 | 6 | 1 | 0 | 100 | 50 | 33 | 17 | 50 | 17 | 0 | 17 | 0 | 0 | 17 | 0 | 17 | 0 | 0 | 0 | 17 | 17 | 17 | 17 | 17 |
| 20s | 21 | 4 | 14 | 86 | 29 | 71 | 0 | 14 | 0 | 0 | 10 | 10 | 5 | 0 | 0 | 5 | 10 | 0 | 0 | 0 | 0 | 0 | 0 | 0 |
| 30s | 37 | 8 | 16 | 84 | 54 | 41 | 5 | 22 | 5 | 5 | 0 | 8 | 8 | 5 | 5 | 0 | 3 | 3 | 0 | 0 | 3 | 5 | 3 | 0 |
| 40s | 86 | 18 | 23 | 77 | 24 | 70 | 6 | 42 | 27 | 19 | 13 | 7 | 6 | 1 | 5 | 5 | 3 | 5 | 2 | 2 | 3 | 3 | 3 | 5 |
| 50s | 142 | 29 | 25 | 74 | 25 | 69 | 5 | 41 | 16 | 18 | 15 | 8 | 6 | 6 | 5 | 3 | 5 | 2 | 6 | 4 | 4 | 3 | 3 | 5 |
| 60s | 131 | 27 | 20 | 79 | 21 | 69 | 8 | 46 | 18 | 18 | 8 | 7 | 7 | 9 | 5 | 4 | 5 | 7 | 6 | 5 | 4 | 3 | 6 | 2 |
| 70s | 63 | 13 | 17 | 83 | 29 | 65 | 6 | 35 | 13 | 11 | 6 | 14 | 13 | 5 | 10 | 14 | 3 | 3 | 3 | 5 | 5 | 3 | 3 | 6 |
| >80 | 6 | 1 | 17 | 83 | 33 | 67 | 0 | 0 | 0 | 17 | 0 | 0 | 0 | 0 | 0 | 0 | 0 | 33 | 0 | 17 | 0 | 0 | 0 | 0 |
| **All Treated** | 490 | 100 | 21 | 79 | 27 | 67 | 6 | 39 | 16 | 15 | 10 | 8 | 7 | 6 | 5 | 5 | 4 | 4 | 4 | 4 | 4 | 3 | 4 | 4 |

Red highlighting denotes relative increased percentages within the ICB Treatment groups, Reported Mutation (RM) groups, and Mutated Gene groups, respectively. Abbreviations: ADCA- Adenocarcinoma; GI - Gastrointestinal; HG - High grade; CRC - Colorectal; OSD - On Study Date; SCC - Squamous Cell Carcinoma.
